# Relationship between photosynthetic phosphorus-use efficiency and foliar phosphorus fractions in tropical tree species

**DOI:** 10.1002/ece3.861

**Published:** 2013-11-06

**Authors:** Amane Hidaka, Kanehiro Kitayama

**Affiliations:** 1 Graduate School of Agriculture, Kyoto UniversityKyoto, 606-8502, Japan

**Keywords:** Leaf traits, nutrient limitation, nutrient use efficiency, photosynthesis, soil nutrients, tropical rain forests

## Abstract

How plants develop adaptive strategies to efficiently use nutrients on infertile soils is an important topic in plant ecology. It has been suggested that, with decreasing phosphorus (P) availability, plants increase photosynthetic P-use efficiency (PPUE) (i.e., the ratio of instantaneous photosynthetic carbon assimilation rate per unit foliar P). However, the mechanism to increase PPUE remains unclear. In this study, we tested whether high PPUE is explained by an optimized allocation of P in cells among P-containing biochemical compounds (i.e., foliar P fractions). We investigated the relationships among mass-based photosynthetic carbon assimilation rate (*A*_mass_), PPUE, total foliar P concentration, and foliar P fractions in 10 tree species in two tropical montane rain forests with differing soil P availability (five species on sedimentary soils and five species on P-poorer ultrabasic serpentine soils) on Mount Kinabalu, Borneo. We chemically fractionated foliar P into the following four fractions: metabolic P, lipid P, nucleic acid P, and residual P. *A*_mass_ was positively correlated with the concentrations of total foliar P and of metabolic P across 10 tree species. Mean *A*_mass_ and mean concentrations of total foliar P and of each foliar P fraction were lower on the P-poorer ultrabasic serpentine soils than on the sedimentary soils. There was a negative relationship between the proportion of metabolic P per total P and the proportion of lipid P per total P. PPUE was positively correlated with the ratio of metabolic P to lipid P. High PPUE is explained by the net effect of a relatively greater investment of P into P-containing metabolites and a relatively lesser investment into phospholipids in addition to generally reduced concentrations of all P fractions. We conclude that plants optimize the allocation of P among foliar P fractions for maintaining their productivity and growth and for reducing demand for P as their adaptation to P-poor soils.

## Introduction

Phosphorus (P) is an essential nutrient in plant metabolism such as photosynthetic carbon assimilation and protein synthesis (Marschner 1995; Sterner and Elser 2002) and frequently limits plant growth and productivity in terrestrial ecosystems (Elser et al. 2007; Vitousek et al. 2010; Harpole et al. 2011). On the other hand, diverse plant species grow on P-poor soils in tropical rain forests (Cleveland et al. 2011) and in ancient landscapes such as in southwestern Australia and in the Cape Region in South Africa (Lambers et al. 2010). Understanding the underlying mechanisms to efficiently acquire P from soils and to efficiently use P for their growth and productivity in such plant species as their adaptation to P-poor soils is an important topic in plant ecology (Aerts and Chapin 2000; Veneklaas et al. 2012). It is suggested that plant species growing on P-poor soils exhibit high photosynthetic P-use efficiency (PPUE) (i.e., the ratio of instantaneous photosynthetic carbon assimilation rate per unit foliar P) (Denton et al. 2007; Hidaka and Kitayama 2009; Lambers et al. 2010). However, the physiological mechanism to increase PPUE still remains unclear (Hidaka and Kitayama 2009, 2011; Lambers et al. 2011, 2012).

PPUE is calculated as the ratio of mass-based maximum photosynthetic carbon assimilation rate (*A*_mass_) per concentration of total foliar P, or as the ratio of area-based maximum photosynthetic carbon assimilation rate (*A*_area_) per area-based content of total foliar P (Small 1972; Hidaka and Kitayama 2009). In general, *A*_mass_ is positively correlated with total foliar P concentration across various plant species (Wright et al. 2004; Hidaka and Kitayama 2009) and is often regulated by decreased foliar P concentration under low P supply (Rao and Terry 1989; Kirschbaum and Tompkins 1990; Jacob and Lawlor 1991; Pieters et al. 2001). Total foliar P concentration consistently decreases, while *A*_mass_ is relatively constant (or decreases rather slightly) with decreasing soil P availability (Hidaka and Kitayama 2009; Cleveland et al. 2011). Hidaka and Kitayama (2009) compared *A*_mass_, total foliar P concentration and PPUE among 340 tree and shrub species along a broad gradient of soil P availability across various biomes and showed that the increase in PPUE with decreasing P availability is caused by the net effects of a relatively greater reduction in total foliar P concentration over a relatively slight reduction in *A*_mass_. On P-poor soils, *A*_mass_ is regulated not only by decreased foliar P concentration but also by increased leaf mass per area (LMA) (Wright et al. 2001; Hidaka and Kitayama 2009), because a greater LMA increases the resistance of CO_2_ diffusion from stomata to mesophyll cells and chloroplasts (Parkhurst 1994; Hanba et al. 1999). On the other hand, the decrease in total foliar P concentration is caused by the sum of reductions of P fractions in cells such as orthophosphate (Pi) and various P-containing biochemical compounds (e.g., nucleic acids, membrane lipids, and metabolites such as sugar phosphates) (i.e., foliar P fractions) (Hidaka and Kitayama 2011; Veneklaas et al. 2012). Therefore, two alternative hypotheses can be logically derived for explaining the underlying mechanism to increase PPUE on P-poor soils. First, PPUE is physiologically increased by optimizing the allocation of P among foliar P fractions to maintain *A*_mass_. Secondly, PPUE is increased by the net effect of reduced concentration of each foliar P fraction irrespective of the allocation of P among foliar P fractions. In both hypotheses, the relative reduction in *A*_mass_ will be smaller than that in total foliar P concentration toward a lower P availability in soils.

During photosynthetic carbon assimilation, Pi and P-containing metabolites are required for various processes such as the production of ATP from ADP, the production and export of triose-P, and the regeneration of ribulose-1,5-bisphosphate (RuBP) (Geiger and Servaites 1994). Therefore, if plants optimize the allocation of P among foliar P fractions for increasing PPUE in line with the former hypothesis, plants will allocate more P into Pi and P-containing metabolites for maintaining *A*_mass_ and will conversely reduce the allocation of P into the other foliar P fractions (e.g., nucleic acids and membrane phospholipids), which are not used for photosynthesis. Most recently, Lambers et al. (2012) suggest that *Banksia* and *Hakea* species (both Proteaeceae), which are dominant on P-poor soils in southwestern Australia, substitute phospholipids with nonphospholipids (i.e., galactolipids and sulfolipids) during leaf development and enhance PPUE by reducing total foliar P concentration. However, it remains unclear whether such a replacement of phospholipids with nonphospholipids increases the allocation of P into Pi and P-containing metabolites for maintaining *A*_mass_ and for increasing PPUE as predicted by our former hypothesis. It also remains unclear whether the reduced concentrations of the major foliar P fractions such as phospholipids and nucleic acids, which are not involved in photosynthesis, are not physiologically but mathematically important for increasing PPUE as predicted by our latter hypothesis. Under low P supply, the major foliar P fractions such as nucleic acids and various P-containing metabolites rather than Pi account for the large proportion in total foliar P, especially when total foliar P concentration is lower than 1 mg g^−1^ (Hidaka and Kitayama 2011; Veneklaas et al. 2012). Therefore, it is necessary to investigate the relationship between PPUE and the allocation of P among the major foliar P fractions for testing the two hypotheses.

In this study, we examined whether high PPUE on P-poor soils is explained by the allocation of P among foliar P fractions by comparing the above two hypotheses. We investigated the relationships among *A*_mass_, PPUE, LMA, and the concentration and proportion of foliar P fractions of tropical tree species in two tropical montane rain forests with differing soil P availability on Mount Kinabalu, Borneo.

## Materials and Methods

### Leaf sampling and measurement of photosynthesis

This study was conducted in two tropical montane rain forests on the southern slopes of Mount Kinabalu (6°05′N, 116°33′E, 4095 m a.s.l.), Sabah, Malaysian Borneo. The study sites were described in detail by Kitayama and Aiba (2002) and Takyu et al. (2002). Two study sites were different in geological substrates (Tertiary sedimentary rock and ultrabasic serpentine rock) and therefore different in soil P availability (0.12, and 0.02 g m^−2^ soluble P extracted with hydrochloric–ammonium fluoride, respectively) (Kitayama et al. 2000; Takyu et al. 2002). The two sites are located at nearly the same altitude (1560 m and 1860 m a.s.l., respectively) and had a comparable climate (mean annual air temperature was 18°C, and mean annual precipitation was 2700 mm) (Kitayama 1992).

In August and September 2006, we measured *A*_area_ of at least two replicate trees each from five tree species, which are relatively dominant and belong to the same family (Fagaceae, Myrtaceae, and Theaceae), at each study site (Table 1). *A*_area_ was measured using a portable open-system infrared gas analyzer (LI6400, Li-Cor, Inc., Lincoln, NE, USA). Measurements were carried out between 0800 and 1200 h on fully expanded healthy sun-exposed leaves at 2–4 m height on each tree. Data were collected with the irradiance, CO_2_ concentration, chamber temperature, and vapor pressure difference between the leaf and air inside the cuvette of the leaf chamber adjusted to approximately 1000 μmol m^−2^ s^−1^, 400 μmol mol^−1^, 25 ± 1°C, and 0.5–1.0 kPa, respectively. Leaves that were measured for CO_2_-assimilation rates were collected, wiped, and punched to form 10-mm-diameter disks for LMA determination, and the rest of leaf samples were immediately freeze-dried for measuring foliar P fractions. For LMA determination, punched-out leaf samples were dried at 70–80°C for 72 h to a constant weight and measured for oven-dried mass. LMA was calculated as oven-dried mass divided by area. *A*_mass_ was calculated from *A*_area_ and LMA.

**Table 1:** Mass-based and area-based photosynthetic assimilation rate (*A*_mass_ and *A*_area_), total foliar P concentration, and photosynthetic P-use efficiency (PPUE) measured for tropical tree species growing on two contrasting soil types on Mount Kinabalu

	*n*	*A*_mass_ (nmol CO_2_ g^−1^ s^−1^)	*A*_area_ (μmol CO_2_ m^−2^ s^−1^)	Total P (mg g^−1^)	PPUE (μmol CO_2_ mol P^−1^ s^−1^)
Ultrabasic site					
*Lithocarpus rigidus* (Fagaceae)	5	28.2	9.1	0.287	3050
*Quercus lowii* (Fagaceae)	2	43.1	9.9	0.374	3570
*Schima brevifoila* (Theaceae)	3	42.5	8.5	0.274	4810
*Syzygium subdecussatum* (Myrtaceae)	3	27.6	7.3	0.276	3090
*Tristaniopsis* cf. *elliptica* (Myrtaceae)	5	41.0	9.7	0.282	4500
Sedimentary site					
*Castanopsis acuminatissima* (Fagaceae)	5	87.6	10.7	0.702	3860
*Lithocarpus clementianus* (Fagaceae)	4	71.7	8.7	0.584	3810
*Schima wallichii* (Theaceae)	4	77.3	9.7	0.418	5730
*Syzygium chrysanthum* (Myrtaceae)	5	55.6	8.0	0.663	2600
*Tristaniopsis clementis* (Myrtaceae)	5	60.1	8.0	0.483	3860
Mean ± SE					
Ultrabasic site	5	36.5 ± 3.5	8.9 ± 0.5	0.30 ± 0.02	3800 ± 360
Sedimentary site	5	70.5 ± 5.8	9.0 ± 0.5	0.60 ± 0.05	4000 ± 500
Significant levels		*P* = 0.001	ns	*P* = 0.001	ns

### Foliar P fractions

We divided foliar P into the following four fractions: metabolic P (including Pi and easily soluble P-containing metabolites such as ATP and sugar phosphates), lipid P (i.e., phospholipids; Hidaka and Kitayama 2011 termed it as structural P), nucleic acid P (i.e., RNA and DNA), and residual P (phosphoproteins and unidentified residue). The fractionation procedure was the same as in Hidaka and Kitayama (2011) based on the following methods as in Kedrowski (1983) and Close and Beadle (2004). Each of the freeze-dried samples was ground after removing their petioles and main veins. Ground sample was homogenized in 12:6:1 CMF (chloroform, methanol, and formic acid) (v/v/v) using a Polytron homogenizer (Multipro Model 395, Dremel Co., Racine, WI, USA; Generator shaft 10φ, SMT Co., Tokyo, Japan), extracted twice with a total of 15 mL CMF and twice with a total of 19 mL 1:2:0.8 CMW (chloroform, methanol, and water) (v/v/v) in a 50-mL centrifuge tube, and was added with 9.5 mL chloroform-washed water. The final solvent ratio was 1:1:0.9 CMW (v/v/v), causing the extract to separate into a lipid-rich organic bottom layer and a sugar- and nutrient-rich aqueous upper layer. This lipid extraction was repeated several times for complete separation (Fraction 1). The residue remaining after the lipid extraction was extracted with 5 mL of 85% (w/v) methanol. The methanol extract was added to the tube containing the aqueous layer from the lipid-phase extraction. This tube was then placed under vacuum for 48 h to remove dissolved chloroform and some of methanol from the aqueous solution. The methanol contained in the residue was also removed. The aqueous layer was added to the tube containing the residue, and after cooling to 4°C, 100% (w/v) trichloroacetic acid (TCA) was added to make to 5% TCA solution. This cold TCA solution was extracted by a second cold extraction with 10 mL 5% (w/v) cold TCA for 1 h, by shaking at 10-min intervals. Aliquots were taken for the analysis of cold TCA soluble P (Fraction 2). The residue remaining from the cold TCA extraction was extracted twice using a total of 35 mL 2.5% (w/v) TCA at 95°C in a hot water bath for 1 h. Aliquots were taken for the analysis of total hot TCA soluble P (Fraction 3). The residue remaining from the hot TCA extraction is Fraction 4. All liquid–solid separations were accomplished by decantation following centrifugation at 1000 *g*. Temperatures of all processes were 20°C unless otherwise stated. In this procedure, lipid P, metabolic P, nucleic acid P, and residual P correspond to Fractions 1, 2, 3, and 4, respectively.

P concentrations in extracts and residues of above procedure, and total P concentrations were measured as in Hidaka and Kitayama (2009). Each sample was digested in 5 mL of concentrated H_2_SO_4_ and 2 mL of 30% H_2_O_2_ at 400°C for 2–3 h. After cooling to 100°C, 30% H_2_O_2_ was added dropwise until the solution became a clear pale yellow color. Digestion was repeated until the solution became clear. The digests were diluted, filtered through Whatman 2 filter paper, and made to 50 mL with deionized water. The concentration of P in the digest was determined using an inductively coupled plasma atomic emission spectrometer (ICPS-7510, Shimadzu Co., Kyoto, Japan). PPUE was calculated as *A*_mass_ divided by total foliar P concentration (μmol CO_2_ mol P^−1^ s^−1^).

### Statistical analyses

We compared means of total foliar P concentration, the concentration and proportion (i.e., the ratio of foliar P fraction per total P) of each foliar P fraction, and LMA between study sites by *t* test, and means of proportion among foliar P fractions within each site by ANOVA with a *post hoc* Tukey HSD test. Relationships of total foliar P concentration to *A*_mass_ and PPUE, and relationships of LMA to total foliar P concentration, *A*_mass,_ and PPUE were analyzed after base 10 log transformation. We also compared values of total foliar P concentration, A_mass,_ and PPUE between our results in this study and the global data in Hidaka and Kitayama (2009). Statistical analyses were performed using R 2.14.1 (http://www.R-project.org).

## Results

### Photosynthetic rates, total foliar P, and photosynthetic P-use efficiency

**Figure 1:**
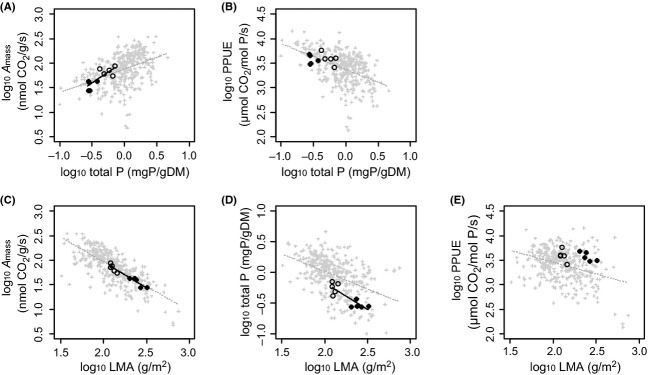
Relationships of total foliar P concentration to mass-based photosynthetic assimilation rate (A_mass_) and photosynthetic P-use efficiency (PPUE), and relationships of leaf mass per area (LMA) to A_mass_, total foliar P concentration and PPUE of 10 tropical tree species on Mount Kinabalu relative to those from global dataset in Hidaka and Kitayama (2009). Symbols: ultrabasic site (solid circles), sedimentary site (open circles), and global dataset (gray crosses). Significant regression lines: Mount Kinabalu (solid) and global dataset (gray dashed).

Mean *A*_mass_ and mean concentration of total foliar P were significantly higher at the sedimentary site than at the ultrabasic site (all *P* < 0.05) (Table 1). However, mean PPUE was not different between two sites (Table 1). *A*_mass_ was positively correlated with total foliar P concentration across 10 species (*R*^*2*^ = 0.63, *P* = 0.006) (Fig. 1A), but not within each site (both *P* > 0.05) (Table S1). There was no significant correlation between PPUE and total foliar P concentration across 10 species in our study (*R*^*2*^ = 0.05, *P* = 0.53) (Fig. 1B).

### Relationships of foliar P fractions to photosynthetic rates and photosynthetic P-use efficiency

**Figure 2:**
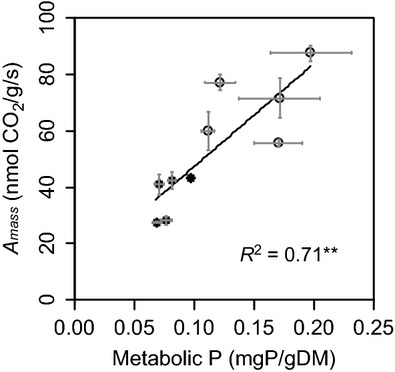
Relationship between mass-based photosynthetic assimilation rate (A_mass_) and the concentration of metabolic P of 10 tropical tree species on Mount Kinabalu. Bars indicate standard error. Symbols are the same as in Fig. 1.

**Figure 3:**
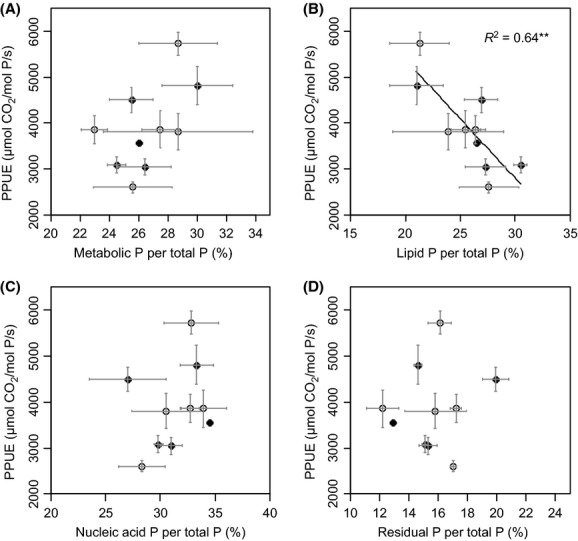
Relationships between photosynthetic P-use efficiency (PPUE) and the proportion of each foliar P fraction of 10 tropical tree species on Mount Kinabalu. Bars indicate standard error. Symbols are the same as in Fig. 1.

**Figure 4:**
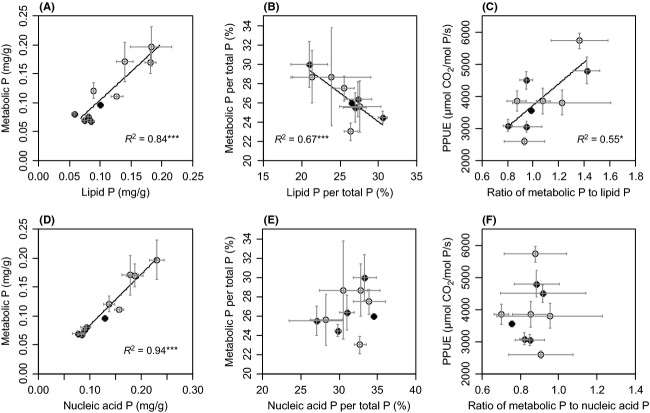
Relationships between metabolic P, lipid P, and nucleic acid P in concentration and proportion, and relationships of photosynthetic P-use efficiency (PPUE) to the ratio of metabolic P to lipid P and to the ratio of metabolic P to nucleic acid P of 10 tropical tree species on Mount Kinabalu. Bars indicate standard error. Symbols are the same as in Fig. 1.

Mean concentration of each foliar P fraction was significantly higher at the sedimentary site than at the ultrabasic site (all *P* < 0.01) (Table 2). On the other hand, mean proportion of each foliar P fraction per total P was not different between two sites (all *P* > 0.05) (Table 2). *A*_mass_ was positively correlated with the concentration of metabolic P (Fig. 2) and of the other foliar P fractions across 10 species (Table S1). The coefficients of determination between *A*_mass_ and metabolic P concentration (*R*^*2*^ = 0.71) and between *A*_mass_ and nucleic acid P concentration (*R*^*2*^ = 0.72) were higher than that between *A*_mass_ and total foliar P concentration (*R*^*2*^ = 0.63). PPUE was negatively correlated with the proportion of lipid P (*R*^*2*^ = 0.64, *P* = 0.006) (Fig. 3B), while tended to increase positively with increasing metabolic P (*R*^*2*^ = 0.29, *P* = 0.11) (Fig. 3A). PPUE was not correlated with the other foliar P fractions (Fig. 3C, D). The concentration of metabolic P was positively correlated with the concentration of lipid P (*R*^*2*^ = 0.84, *P* < 0.001) (Fig. 4A) and nucleic acid P (*R*^*2*^ = 0.94, *P* < 0.001) (Fig. 4D). On the other hand, the proportion of metabolic P was negatively correlated with the proportion of lipid P (*R*^*2*^ = 0.67, *P* = 0.004) (Fig. 4B), but not with the other foliar P fractions (*P* > 0.05) (Fig. 4E, Table S1). As a consequence, PPUE was positively correlated with the ratio of metabolic P to lipid P (*R*^*2*^ = 0.55, *P* = 0.01) (Fig. 4C), but not with the ratio of metabolic P to nucleic acid P (*P* > 0.05) (Fig. 4F).

**Table 2:** Mean values ± SE in the concentrations (mg P g^−1^ dry matter) and proportions (%; the concentration of each foliar P fraction per total P concentration) of foliar P fraction of tropical tree species growing on two contrasting soil types on Mount Kinabalu. Pairwise significant differences at *P* < 0.05 among the proportion of foliar P fractions within each site are shown in different letters

	Ultrabasic site (*n* = 5 species)	Sedimentary site (*n* = 5 species)	Significant levels
Metabolic P			
Concentration (mg g^−1^)	0.078^b^ ± 0.005	0.154^a^ ± 0.016	*P* = 0.002
Proportion (%)	26.5 ± 0.9	26.7 ± 1.1	ns
Lipid P			
Concentration (mg g^−1^)	0.079^b^ ± 0.007	0.144^a^ ± 0.018	*P* = 0.009
Proportion (%)	26.4 ± 1.5	24.9 ± 1.1	ns
Nucleic acid P			
Concentration (mg g^−1^)	0.094^b^ ± 0.009	0.179^a^ ± 0.016	*P* = 0.002
Proportion (%)	31.1 ± 1.3	31.6 ± 1.0	ns
Residual P			
Concentration (mg g^−1^)	0.045^b^ ± 0.002	0.088^a^ ± 0.007	*P* < 0.001
Proportion (%)	15.6 ± 1.2	15.7 ± 0.9	ns

### Relationships of leaf mass per area to photosynthetic rates, photosynthetic P-use efficiency, and foliar P fractions

Mean LMA was almost twice greater at the ultrabasic site (251 ± 20 g m^−2^) than at the sedimentary site (129 ± 4 g m^−2^) (*P* < 0.001). LMA was negatively correlated with *A*_mass_ (*R*^*2*^ = 0.92, *P* < 0.001) (Fig. 1C) and total foliar P concentration (*R*^*2*^ = 0.71, *P* = 0.002) (Fig. 1D), but not with PPUE (*R*^*2*^ = 0.09, *P* = 0.41) (Fig. 1E). Our study species at both sites had higher values of PPUE and lower values of total P concentration in comparison with the values at corresponding LMA in the global dataset (Fig. 1D, E). LMA was negatively correlated with the concentration of each foliar P fraction (Table S1), but not with the proportion of each foliar P fraction (Table S1).

## Discussion

Our aim in this study was to examine whether high PPUE on P-poor soils is explained by the allocation of P among foliar P fractions by comparing two hypotheses; first, PPUE is physiologically increased by optimizing the allocation of P among foliar P fractions to maintain *A*_mass_; and secondly, PPUE is increased by the net effect of reduced concentration of each foliar P fraction irrespective of the allocation of P among foliar P fractions. Our result that PPUE positively correlates with the ratio of metabolic P to lipid P (Fig. 4C) supports the former hypothesis and also explains the mechanism to cause the interspecific variation of PPUE within a forest. This result implies that a relatively greater amount of P is allocated into metabolic P for maintaining *A*_mass_, while sacrificing lipid P. It must be noted that Hidaka and Kitayama (2011) earlier suggest that the mean allocation of P among foliar P fractions (i.e., proportion) is invariable between the same two forests as the present paper, in line with the result of the present study (Table 2). We, however, point out that there is interspecific variation in P fractions and negative relationships between lipid P and metabolic P in proportion to total P (Fig. 4B), although there is no trade-off between lipid P and metabolic P in concentration (Fig. 4A). Because *A*_mass_ is weakly positively correlated with the proportion of metabolic P and negatively with the proportion of lipid P (Table. S1), the interspecific variation of P fractions represents a meaningful plant adaptation. Lambers et al. (2012) found that Proteaceae species growing on P-poor soils in southwestern Australia reduce the concentration of lipid P via replacing phospholipids by nonphospholipids (i.e., galactolipids and sulfolipids) during leaf development. Such a replacement of phospholipids by nonphospholipids was found in several model plants in P-starvation experiments (Dörmann and Benning 2002; Tjellström et al. 2008). The substitution of phospholipids with nonphospholipids may also be the case in our sites. On the other hand, it still remains unclear that increased allocation of P into metabolic P is actually used for photosynthesis. Further investigation is required for testing whether the increased proportion of metabolic P actually contributes to maintaining *A*_mass_ and to increasing PPUE on P-poor soils.

Our results generally support the former hypothesis that PPUE is physiologically increased by optimizing the allocation of P among foliar P fractions to maintaining *A*_mass_. However, our results could not reject the latter hypothesis, because low values of total foliar P concentration in P-poorer ultrabasic site (0.27 to 0.37 mg g^−1^) are caused by the net effect of reduced concentrations of metabolic P as well as lipid P and nucleic acid P (Table 2) in line with the earlier findings (Mulligan 1988; Close and Beadle 2004; Hidaka and Kitayama 2011; Veneklaas et al. 2012). In this study, we found that the concentrations of metabolic P and nucleic acid P are strongly and positively correlated with each other (Fig. 4D) and are both similarly positively correlated with *A*_mass_ and negatively with LMA (Table S1). Studies of ecological stoichiometry have suggested that a great amount of nucleic acid P is included in P-rich ribosomal RNA (Sterner and Elser 2002) and that higher rates of plant growth require a greater investment in ribosomal RNA to produce the proteins required for growth (Sterner and Elser 2002; Ågren 2004; Niklas et al. 2005; Reef et al. 2010). Tree species with higher growth rates and a fast turnover of leaves (i.e., lower LMA) contain more proteins in their leaves than tree species with slower growth rates and a slow turnover of leaves (i.e., higher LMA) do (Villar and Merino 2001; Villar et al. 2006). Therefore, the concentration of nucleic acid P can be positively correlated with the rates of protein synthesis and the division of cells in leaves, and negatively with leaf life span and LMA. Because *A*_mass_ is often positively correlated with plant growth rates and respiration rates (Poorter et al. 1990; Walters and Reich 1996; Wright et al. 2004), there must be a positive correlation between the concentration of metabolic P (i.e., for photosynthesis) and the concentration of nucleic acid P (i.e., for protein synthesis) as we found. These findings suggest that tree species growing on P-poor soils can reduce the demand for metabolic P and nucleic acid P in association with their slow growth rate and long life span leaves (i.e., greater LMA), and as a result, reduce total foliar P. The reduced total foliar P increases PPUE, partly in line with the latter hypothesis. Although we could not reject the latter hypothesis, our results are in favor of the former hypothesis: the increased PPUE via the net effect of reduced concentration of each foliar P fraction is caused by optimizing the allocation of P among foliar P fractions with balancing the maintenance of photosynthesis, the synthesis of proteins, and the division of cells.

In conclusion, high PPUE is explained by the net effect of a relatively greater investment of P into P-containing metabolites and a relatively lesser investment into phospholipids in addition to generally reduced concentrations of all P fractions. Plants optimize the allocation of P among foliar P fractions for maintaining their productivity and growth (i.e., maintaining *A*_mass_ and enhancing PPUE) and for reducing the demand for P (i.e., reducing total foliar P concentration) as their adaptation to P impoverishment.

## Supporting Information

**Table S1** Pearson's correlation between the concentrations of foliar P fractions, total P concentration, mass-based photosynthetic assimilation rate (*A*_mass_), photosynthetic P-use efficiency (PPUE), and leaf mass per area (LMA) (above diagonal) and between the proportions of foliar P fractions per total P, total P concentration, *A*_mass_, PPUE, and LMA (below diagonal) of tropical tree species on two contrasting soil types on Mount Kinabalu.
