# Supplementary material for: Relationship between photosynthetic phosphorus-use efficiency and foliar phosphorus fractions in tropical tree species
Source: Ecol Evol. 2013 Nov 6;3(15):4872–80. doi: 10.1002/ece3.861 (PMC3892354; doi:10.1002/ece3.861)
Supplement: Supplementary file 1 [file ece30003-4872-SD1.doc]

**Table S1** Pearson’s correlation between the concentrations of foliar P fractions, total P concentration, mass-based photosynthetic assimilation rate (*A*_mass_), photosynthetic P-use efficiency (PPUE) and leaf mass per area (LMA) (above diagonal) and between the proportions of foliar P fractions per total P, total P concentration, *A*_mass_, PPUE and LMA (below diagonal) of tropical tree species on two contrasting soil types on Mount Kinabalu.

|  | Metabolic P | Lipid P | Nucleic acid P | Residual P | total P | *A*_mass_ | PPUE | LMA |
| --- | --- | --- | --- | --- | --- | --- | --- | --- |
| Metabolic P | Serpentine  Sedimentary  Both | 0.416  0.820  0.916*** | 0.964**  0.907*  0.970*** | 0.070  0.494  0.859** | 0.881*  0.929*  0.978*** | 0.604  0.389  0.842** | 0.066  -0.507  -0.125 | -0.364  -0.193  -0.791** |
| Lipid P | -0.967**  -0.731  -0.816** | Serpentine  Sedimentary  Both | 0.617  0.892*  0.934*** | 0.351  0.747  0.891*** | 0.777  0.974**  0.972*** | -0.152  -0.056  0.649* | -0.693  -0.849  -0.426 | 0.311  0.287  -0.649* |
| Nucleic acid P | 0.516  0.086  0.309 | -0.466  -0.427  -0.462 | Serpentine  Sedimentary  Both | 0.055  0.404  0.839** | 0.945*  0.942*  0.980*** | 0.436  0.384  0.846** | -0.161  -0.559  -0.145 | -0.241  -0.154  -0.803** |
| Residual P | -0.265  -0.446  -0.342 | 0.149  0.121  0.127 | -0.923*  -0.586  -0.790** | Serpentine  Sedimentary  Both | 0.348  0.674  0.914*** | 0.414  -0.608  0.675* | 0.201  -0.983*  -0.231 | -0.093  0.646  -0.779** |
| total P | -0.168  0.037  0.041 | 0.054  0.628  -0.060 | 0.605  -0.271  0.097 | -0.529  -0.542  -0.209 | Serpentine  Sedimentary  Both | 0.425  0.124  0.794** | -0.214  -0.750  -0.229 | -0.163  0.102  -0.783** |
| *A*_mass_ | 0.484  0.671  0.341 | -0.679  -0.567  -0.505 | 0.319  0.733  0.346 | 0.030  -0.884*  -0.200 | 0.425  0.124  0.794** | Serpentine  Sedimentary  Both | 0.793  0.543  0.385 | -0.847  -0.860  -0.909*** |
| PPUE | 0.650  0.467  0.537 | -0.781  -0.923*  -0.798** | -0.051  0.645  0.302 | 0.378  -0.109  0.123 | -0.214  -0.750  -0.229 | 0.793  0.543  0.385 | Serpentine  Sedimentary  Both | -0.801  -0.583  -0.327 |
| LMA | -0.495  -0.639  -0.228 | 0.618  0.668  0.484 | -0.282  -0.682  -0.230 | 0.040  0.644  0.027 | -0.163  0.102  -0.783** | -0.847  -0.860  -0.909*** | -0.801  -0.583  -0.327 | Serpentine  Sedimentary  Both |

Notes: Significant levels: *, *P* < 0.05; **, *P* < 0.01; ***, *P* < 0.001
